# 2103. Comparison of Antibiograms Across Solid Organ Transplant Services Within a Medical Center

**DOI:** 10.1093/ofid/ofac492.1725

**Published:** 2022-12-15

**Authors:** Annie Kim, Conan MacDougall

**Affiliations:** University of California, San Francisco Medical Center, San Francisco, California; University of California San Francisco, San Francisco, CA

## Abstract

**Background:**

Antibiograms summarize localized antimicrobial susceptibilities and are used to guide empiric antibiotic therapy. Antibiograms for subpopulations may offer more meaningful clinical data, especially in immunocompromised hosts who may be at higher risk for multidrug-resistant organisms. However, population-specific antibiograms are uncommon. The purpose of this study is to evaluate whether service-specific antibiograms provide more useful information than hospital-wide antibiograms.

**Methods:**

This is a retrospective, single-center study that included bacterial isolates from all body sites collected between 2017-2020. Antibiograms were created in accordance with the Clinical and Laboratory Standards Institute guidelines, with susceptibilities reported as a percentage and a 95% confidence interval. A combined solid organ transplant (SOT) antibiogram and individual antibiograms based on the transplanted organ (heart, lung, liver, and kidney) were compared to a hospital antibiogram with a difference of ≥ 10% considered clinically significant.

**Figure:**
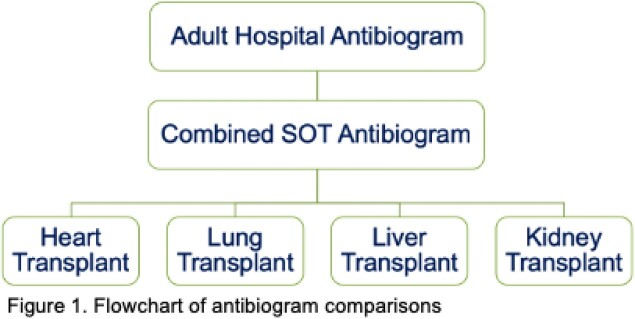

**Results:**

In the combined SOT antibiogram, *Escherichia coli*, *Klebsiella aerogenes*, and *Klebsiella pneumoniae* susceptibilities were lower for antibiotics such as ceftriaxone, ceftazidime, and ciprofloxacin compared to the hospital antibiogram. Overall susceptibilities for *Pseudomonas aeruginosa* in the SOT antibiogram were comparable to that of the hospital antibiogram; however piperacillin-tazobactam susceptibilities were substantially lower among lung and heart transplant patients. Among *Staphylococcus aureus* isolates, clindamycin susceptibilities were similar between the SOT antibiogram and hospital antibiogram. This was a result of lower susceptibilities among lung transplant patients, offset by greater susceptibility in liver, kidney, and heart transplant patients.

**Figure:**
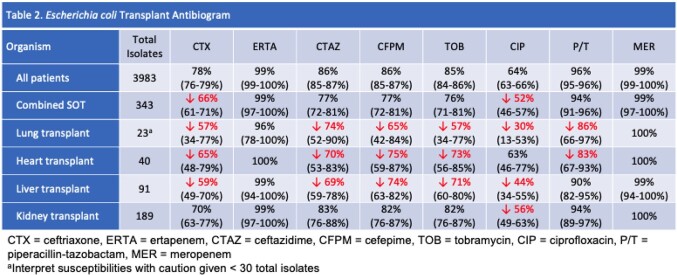

**Figure:**
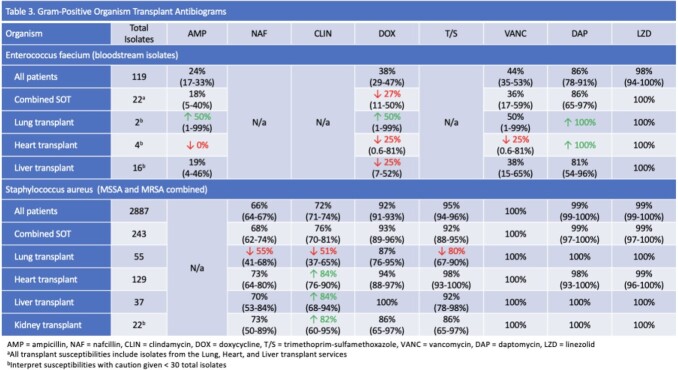

**Conclusion:**

Clinically significant differences were seen in susceptibilities for several antibiotics against gram-negative and gram-positive pathogens in various transplant antibiograms. The combined SOT antibiogram minimized substantial between-service differences. Through population-specific antibiograms, providers may be able to improve empiric antibiotic therapy selection for their patient population.

**Disclosures:**

**Conan MacDougall, PharmD, MAS**, Merck: Advisor/Consultant.

